# The Sea Peoples, from Cuneiform Tablets to Carbon Dating

**DOI:** 10.1371/journal.pone.0020232

**Published:** 2011-06-08

**Authors:** David Kaniewski, Elise Van Campo, Karel Van Lerberghe, Tom Boiy, Klaas Vansteenhuyse, Greta Jans, Karin Nys, Harvey Weiss, Christophe Morhange, Thierry Otto, Joachim Bretschneider

**Affiliations:** 1 EcoLab (Laboratoire d'Ecologie Fonctionnelle et Environnement), Université Paul Sabatier-Toulouse 3, Toulouse, France; 2 EcoLab (Laboratoire d'Ecologie Fonctionnelle et Environnement), CNRS, Toulouse, France; 3 Center for Archaeological Sciences, Katholieke Universiteit Leuven, Heverlee, Belgium; 4 Near Eastern Studies, Faculteit Letteren, Katholieke Universiteit Leuven, Leuven, Belgium; 5 Mediterranean Archaeological Research Institute, Vrije Universiteit Brussel, Brussels, Belgium; 6 Environmental Studies Program, Yale University, New Haven, Connecticut, United States of America; 7 CEREGE, UMR CNRS 6635, Aix-Marseille Université, Université de Provence, Europôle de l'Arbois, Aix-en-Provence, France; Universidad Autonoma de Barcelona and University of York, Spain

## Abstract

The 13^th^ century BC witnessed the zenith of the Aegean and Eastern Mediterranean civilizations which declined at the end of the Bronze Age, ∼3200 years ago. Weakening of this ancient flourishing Mediterranean world shifted the political and economic centres of gravity away from the Levant towards Classical Greece and Rome, and led, in the long term, to the emergence of the modern western civilizations. Textual evidence from cuneiform tablets and Egyptian reliefs from the New Kingdom relate that seafaring tribes, the Sea Peoples, were the final catalyst that put the fall of cities and states in motion. However, the lack of a stratified radiocarbon-based archaeology for the Sea People event has led to a floating historical chronology derived from a variety of sources spanning dispersed areas. Here, we report a stratified radiocarbon-based archaeology with anchor points in ancient epigraphic-literary sources, Hittite-Levantine-Egyptian kings and astronomical observations to precisely date the Sea People event. By confronting historical and science-based archaeology, we establish an absolute age range of 1192–1190 BC for terminal destructions and cultural collapse in the northern Levant. This radiocarbon-based archaeology has far-reaching implications for the wider Mediterranean, where an elaborate network of international relations and commercial activities are intertwined with the history of civilizations.

## Introduction

The late 13^th^ century BC was a time of uncertainty and conflict for peoples and polities of the Aegean and Eastern Mediterranean [1], [2], [3]. Written evidences relate a weakening of central administrations [2], an erosion of political powers [4], [5], and a widespread food shortage [6]–[8] underpinned by devastating drought [9], [10]. This sequence of high-magnitude events led to the Sea People event and to the collapse of the ancient Mediterranean world around 1200 BC [1], [3], [5]. Cuneiform tablets foreshadowing the fall of the thriving coastal city Ugarit [2], and reliefs from Ramses III's mortuary temple at Medinet Habou depicting a chaotic scene of boats and warriors entwined in battle in the Nile delta [11], attest that vast movements of seafaring and inland tribes, the Sea Peoples [12] (or Land and Sea Peoples), lie at the heart of changes for this period.

**Figure 1:**
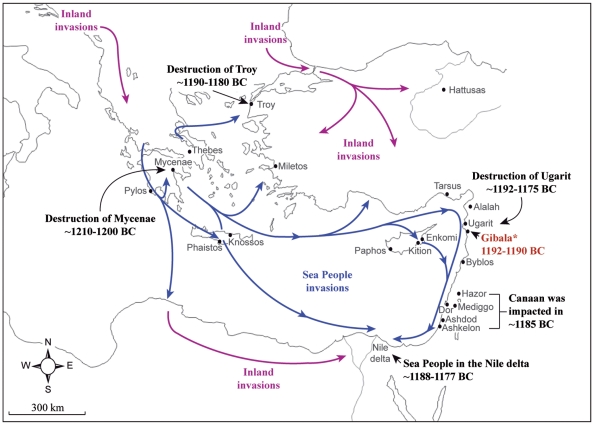
Map of the Sea People invasions in the Aegean Sea and Eastern Mediterranean at the end of the Late Bronze Age (blue arrows). Some of the major cities impacted by the raids are denoted with historical dates. Inland invasions are represented by purple arrows.

The Sea Peoples were seaborne foes [13], [14], [15] from different origins [6], [12]. They launched a combined land-sea invasion (Fig. 1) that destabilized the already weakened power base of empires and kingdoms of the old world, and attempted to enter or control the Egyptian territory [11]. The Sea Peoples symbolize the last step of a long and complex spiral of decline in the ancient Mediterranean world [2], [3], [4], [5]. Cuneiform tablets from Ugarit provide an impressive glimpse of the frantic preparations which the city and her neighbours pursued, in vain, to ward against the invasions [2]. The destructive operations of the Sea Peoples are later narrated by Ramses III who claims on his mortuary temple: “*No land could stand before their arms: from Hatti, Qode, Carchemish, Arzawa, and Alashiya on, being cut off (destroyed) at one time*” [16]. Within the conventional view, the Sea Peoples are linked in history to the collapse of the Late Bronze Age cultures [4], and 1200 BC stands as a symbolic date in human civilization.

**Figure 2:**
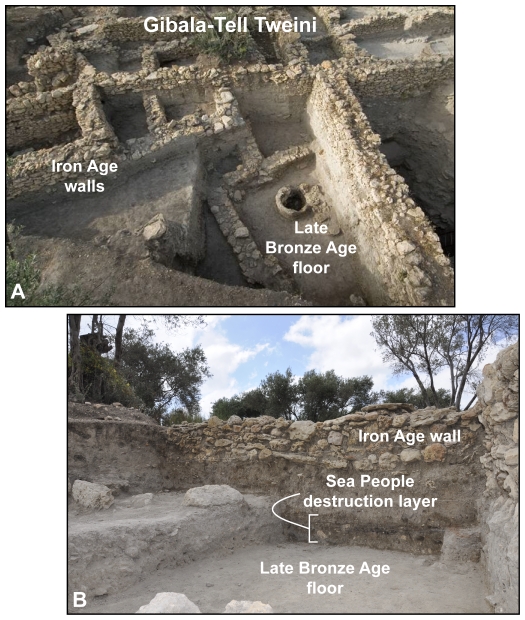
The harbour town Gibala-Tell Tweini and the Sea People destruction layer. The picture A is an aerial view of the eastern part of the excavation field A at Gibala-Tell Tweini. The picture B shows the Sea People destruction layer with ashes, stone rubbles from fallen walls, and ceramic fragments.

Whereas the Sea People event constitutes a major turning point in ancient world history, attested by both written and archaeological (*e.g.* Ugarit, Enkomi, Kition, Byblos) evidence, our knowledge of when these waves of destructions occurred rests on translation of cuneiform tablets preceding the invasions (*terminus ante quem*) and on Ramses III's reign (*terminus post quem*). Here, we report the first absolute chronology of the invasion from a rare, well-preserved Sea People destruction layer (Fig. 2) from a Levantine harbour town of the Ugarit kingdom. The destruction layer contains remains of conflicts (bronze arrowheads scattered around the town, fallen walls, burnt houses), ash from the conflagration of houses, and chronologically well-constrained ceramic assemblages fragmented by the collapse of the town. This stratified radiocarbon-based archaeology, with anchor points in ancient epigraphic-literary sources, Hittite-Levantine-Egyptian kings and astronomical observations, was used to precisely date the Sea People invasion in northern Levant, a decisive episode in a long-term collapse of the ancient Eastern Mediterranean world. By confronting historical and science-based archaeology, the data offer the first firm chronology for this key period in human society.

### The harbour town Gibala, Ugarit Kingdom

Sample collection for radiocarbon (^14^C) dating of the Sea People event was performed at the harbour town Gibala [17], [18], [19], a thriving Levantine trade center located at the southernmost edge of the powerful Ugarit kingdom [20], [21]. Gibala (present Tell Tweini, 35°22′17.93″N, 35°56′12.60″E; elevation: 19 to 27 meters above sea level ) was integrated into a large network of long-distance trading and cultural exchanges between the Aegean, Cyprus, the Levant, Egypt and Western Asia. Direct access from the Mediterranean to the Syrian heartland, Anatolia, and Mesopotamia afforded the ports of the Ugarit kingdom their wealth. This strategic position sets the chronology obtained for the destruction of Gibala by the Sea Peoples in a Mediterranean-wide perspective for the end of the Late Bronze Age (Fig. 1), extending over trade routes that crossed both land and sea. The place name Gibala appears on two 14^th^ century BC cuneiform tablets from Ugarit [17]. The written Bronze Age sources or epigraphic finds for Gibala cease as soon as Ugarit was destroyed by the Sea Peoples. Gibala-Tell Tweini is a large multi-period site of ∼12 hectares (Fig. 2), which was occupied from Early Bronze Age IV (2400 BC) to Iron Age III (500 BC). During the past 11 years, large-scale excavations have elucidated a thriving Bronze Age city that remained occupied during the Iron Age, with only short periods of abandonment despite a massive fire at the end of the Early Iron Age [short-lived sample: 2845±35 ^14^C years before the present (^14^C yr BP from AD 1950)] (Fig. S1) (Supporting Information S1). Gibala is a rare coastal settlement, alongside Tell Kazel, Ras Ibn Hani and Ras el-Bassit, with reoccupations after the Sea People event. A stable water supply, provided by the northern Rumailiah River and the southern Ain Fawar spring-complex, may explain resettlements on the Gibala's alluvial plain since the Early Iron Age [10].

## Results and Discussion

**Figure 3:**
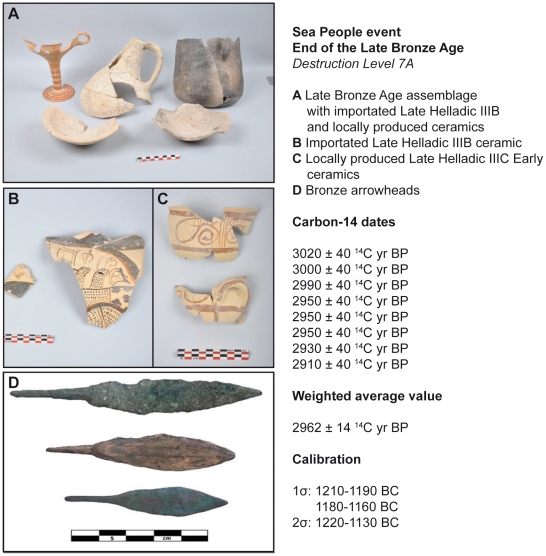
Gibala, Ugarit Kingdom: bronze arrowheads and typical ceramic assemblage for the end of the Late Bronze Age and the Sea People event in the Aegean and Eastern Mediterranean. Ceramics and arrowheads were retrieved from the destruction Level 7A. The ^14^C weighted average value and calibrations provide a robust chronological framework for the Sea People event.

**Figure 4:**
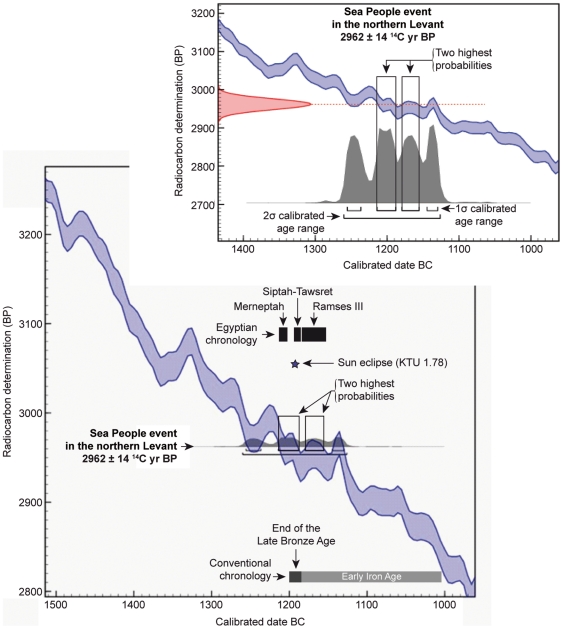
Radiocarbon dating result placed in stratigraphic order on the calibration curve. The horizontal scale is in historical years BC. The vertical scale is in conventional radiocarbon years BP.

About 300 years after a first conflagration (short-lived sample: 3190±40 ^14^C yr BP) (Table 1) (Supporting Information S1), the site was abandoned following a severe destruction at the end of the Late Bronze Age (Fig. 2). The widespread ash layer, termed Level 7A, contained rich finds (Fig. 3) including bronze arrowheads resulting from fights in the harbour town before its destruction, and a large variety of Mycenaean (Late Helladic IIIB), local Late Helladic IIIC Early, and Late Cypriot IIC ceramics (e.g. White slip II) highly significant for the Sea People event in the ancient Mediterranean world [17], [18]. This ash layer is nearly synchronous with the Sea People destruction of Ugarit, and other northern Levantine coastal sites such as Ras Ibn Hani, Ras el-Bassit, Tell Kazel, and Tell Sukas [17]. Short-lived samples and young branches found in the destruction debris from eight key loci (Fig. S2) were dated by accelerator mass spectrometry (AMS). The samples pooled in the matrix (Table 1) are statistically the same at the 95% confidence level using a Chi-square (χ^2^) test (sample key 7, where *T* = 6.09<14.1). The weighted average date (2962±14 ^14^C yr BP) gives a 1 sigma (σ) calibrated age range of 1215–1190 BC with 34.3% relative probability and another age range of 1180–1160 BC with 26% relative probability, using Calib-Rev. 6.0.1 [22] and Oxcal 4.1 [23] with IntCal09. Calibrated age ranges in details at 60.3% of the 100% dating probability (Fig. 4). Hence, there are two chronological possibilities for the calibrated date of the destruction Level 7A, between the end of the 13^th^ century and the beginning of the 12^th^ century BC or the first half of the 12^th^ century BC. By contrasting historical-archaeological and radiocarbon-based data sets, the best candidate for the destruction date of the harbour town is the Sea People invasion. Their presence immediately after the destruction of Gibala is indicated by the material culture of the new settlements on the Tell namely the appearance of Aegean-type architecture, locally-made Mycenaean IIIC Early pottery, hand-made burnished pottery, and Aegean-type loam-weights. These materials, also known from Philistine settlements [24], are cultural markers of foreign settlers, most probably the Sea Peoples.

**Table 1:** Detail of the radiocarbon calibrated ages.

					Calibrated dates BCE		
Code	Material	Species	AMS ^14^C yr BP	δ^13^C, ‰	1σ - 68%	2σ - 95%	
Beta-281588	Charcoal	*Pyrus syriaca*	3300±40	−25,4	1620-1520	1680-1500	Middle-Late Bronze Age*Level 7D*
Beta-281571	Charcoal	*Quercus* sp.	3290±40	−25	1620-1510	1670-1490	
Beta-281577	Charcoal	deciduous *Quercus*	3290±40	−25,4	1620-1510	1670-1490	
Beta-281589	Charcoal	*Quercus* sp.	3280±40	−24,8	1610-1500	1650-1460	
Beta-281578	Charcoal	Evergreen *Quercus*	3240±40	−26,3	1530-1460	1610-1430	
Beta-281573	Charcoal	*Cedrus libani*	3240±40	−24,9	1530-1460	1610-1430	
Beta-281584	Charred seeds	*Lens culinaris*	3190±40	−23,5	1500-1420	1520-1400	
Beta-281586	Charcoal	deciduous *Quercus*	3190±40	−24,8	1500-1420	1520-1400	
Beta-281587	Charcoal	*Quercus* sp.	3170±40	−26,6	1490-1410	1510-1390	
Beta-281585	Charred stones	*Olea europaea*	3020±40	−21,2	1320-1250	1400-1130	**Sea People event** *Destruction Level 7A*
Beta-281582	Charred stones	*Olea europaea*	3000±40	−21,5	1310-1200	1380-1120	
Beta-281576	Charred stones	*Olea europaea*	2990±40	−24,3	1300-1190	1330-1120	
Beta-281572	Charred stones	*Olea europaea*	2950±40	−25,9	1260-1120	1300-1020	
Beta-281590	Charred seeds	*Brassica oleracea*	2950±40	−22,4	1260-1120	1300-1020	
Beta-281591	Charred stones	*Olea europaea*	2950±40	−27,7	1260-1120	1300-1020	
Beta-281579	Charcoal	*Olea europaea*	2930±40	−19,8	1210-1050	1270-1010	
Beta-281570	Charred stones	*Olea europaea*	2910±40	−25,1	1140-1020	1260-1000	
Beta-281581	Charcoal	deciduous *Quercus*	2900±40	−24,2	1130-1010	1220-980	Early Iron Age*Level 6E*
Beta-281574	Charcoal	deciduous *Quercus*	2900±40	−26,5	1130-1010	1220-980	
Beta-281568	Charcoal	deciduous *Quercus*	2880±40	−23,7	1120-1000	1200-930	
Poz-25443	Charcoal	deciduous *Quercus*	2880±35	Nd	1120-1010	1130-970	
Beta-281583	Charcoal	deciduous *Quercus*	2870±40	−25	1120-1000	1140-920	
Beta-281569	Charcoal	deciduous *Quercus*	2870±40	−23,8	1120-1000	1140-920	
Beta-281580	Charcoal	deciduous *Quercus*	2860±40	−25,9	1080-980	1130-920	
Poz-25442	Charred stones	*Olea europaea*	2845±35	Nd	1050-970	1120-920	
Beta-281575	Charcoal	deciduous *Quercus*	2810±40	−25,4	1010-910	1050-890	
Poz-26396	Charcoal	deciduous *Quercus*	2780±35	Nd	980-900	1010-840	

The internal consistency of the typical imported ceramic assemblage found in the destruction layer (Fig. 3) and the ^14^C dating results (Fig. 4) indicate that Level 7A is a secured layer to date the Sea People event in northern Levant. The Late Helladic IIIB-IIIC (Early) and Late Cypriot IIC-IIIA transitions respectively dated in mainland Greece to 1210–1175 BC [25] and in Cyprus to 1220–1190 BC [26]–[27], are markers for the end of the palatial civilization in both the Aegean and Cyprus. All the Mycenaean imports ended in the region of the Ugarit kingdom with its destruction by the Sea Peoples who interrupted trade routes. Neutron activation analyses suggest that the Late Helladic IIIB vessels found at Gibala until the 13^th^ century BC originate from the northern Peloponnese area [19]. The Late Cypriot IIC ceramics were imported from Cyprus.

The ^14^C dating results of Gibala are closely matched with historical dates suggested by Egyptologists for the reign of the Pharaoh Merneptah (1213–1203 BC), and Pharaoh Siptah-Queen Tawsret (1194–1186 BC) [28] (Fig. 4). The cuneiform tablets found in the ruins of Ugarit and Ras Ibn Hani (tablets RS 17.226, RS 18.038, RS 88.2158, RS 20.212, RS 20.238, RS 34.129, RS 19.011) are evidence for the rich correspondence between the last king of Ugarit (Ammurapi; first regnal year 1215 BC), the last king of Hatti (Suppiluliuma II; first regnal year 1210 BC), the king of Carchemish (Talmi-Tešub; first regnal year 1220 BC), and Pharaoh Merneptah [2]. These letters demonstrate that Ugarit was still a kingdom at the very end of the 13^th^ century BC but also relate that its territory was threatened by seaborne and land invasions (Fig. 1). The cuneiform tablet RS 86.2230 [29], sent by the Egyptian Bay (1194–1190 BC), the Great Chancellor of the Pharaoh Siptah-Queen Tawsret, to the last king of Ugarit provides the final evidence of a living kingdom at the dawn of the 12^th^ century BC. A document from Egypt relating the execution of Bay as a traitor in Siptah's regnal year 5, states that the cuneiform tablet RS 86.2230 must have been written before an historical date of 1190 BC [30].

The radiocarbon results, in relation to archaeological and historical data, lead us to propose a date of 1194–1190 BC for the Sea People event in the northern Levant. This radiocarbon-based archaeological date can be refined with the astronomical observation related on the cuneiform tablet KTU 1.78 (RS 12.061) found among the ruins of Ugarit. The sun eclipse depicted on the cuneiform tablet KTU 1.78 was dated to the 21 January 1192 BC [31], suggesting that the destruction of the city and the fall of the kingdom occurred after this observation. The date of 1192–1190 BC for the Sea People invasions in the northern Levant, and the end of the ancient Eastern Mediterranean world fits the radiocarbon, historical, archaeological and astronomical data.

Our research suggests that the traditional Egyptian date for the decline of the ancient Mediterranean world, based on sources from Ramses III's reign, only corresponds to the final part of a more complex and longer-term event that intensified after 1215 BC with the first written evidence of food shortage [6], [7], [8]. According to the Great Harris Papyrus and to the scenes of naval and land battles depicted at Medinet Habu (Thebes, Upper Egypt), Ramses III defeated the Sea Peoples during the 8^th^ year of his reign [11]. The first regnal year of Ramses III is variously dated in the literature [28], [32], [33], giving historical dates of 1176 BC, 1179 BC, and a radiocarbon-based date of 1188–1177 BC for the Sea People invasions in the Nile delta. According to the 1192–1190 BC proposed date, the civilizations of the Aegean and Eastern Mediterranean were devastated long before the war the Sea Peoples waged against Ramses III's army.

By a combined use of radiocarbon, archaeological and historical data, the first firm date of 1192–1190 BC is proposed for the terminal destruction and disintegration of Late Bronze Age societies in the Northern Levant. The collapse caused by the Sea Peoples marks a historical watershed and from these crisis years arose a new world. Later, the Greeks narrated and heroised this period with the myths and stories on the fall of Troy (Homer's Iliad and Odyssey). Some of the Sea Peoples, the Philistines [12], received a significant recognition in Biblical texts [34], and the name Palestine derives from these settlers.

## Materials and Methods

Quality control on sample collection for ^14^C measurements was undertaken during excavations. Only samples originating in reliable contexts with clear association to meaningful ceramic assemblages and occupation levels were used. Samples were selected from primary contexts in May 2010, with an emphasis placed on short-lived samples (seeds or olive stones) (Fig. S2) and young branches (Fig. S3). All botanical macro-remains were sampled from the Middle-Late Bronze Age to the Iron Age II layers and subsequently determined using optical and scanning electron microscopes (Fig. S2). A total of 24 secured samples were dated by Beta Analytic (Miami, Florida) and Poznan Radiocarbon Laboratory (Poznan) using standard accelerator mass spectrometry ^14^C. An acid/alkali/acid pretreatment was performed and the radiocarbon ages were measured. The ^13^C/^12^C ratio and the conventional radiocarbon age were provided for each sample with two-sigma calendar calibration results. The weighted average value for the fire event termed Level 7A results from multiple measurements of the same ash layer from different key loci. The samples pooled in the matrix were statistically tested at a 95% confidence level using a χ^2^ test. The weighted average value was calibrated using Calib. Rev. 6.0.1 [22] and Oxcal 4.1 [23].

## Supporting Information

Figure S1 **Gibala-Tell Tweini: storage jars found in the Early Iron Age destruction layer.** The carbon-14 dating results provide a chronological framework for the Early Iron Age in the Northern Levant.
(TIF)

Figure S2 **Scanning electron microscopy pictures showing the Sea People event burnt macro-remains of short-lived samples (** ***Olea europaea*** **,** ***Brassica oleracea*** **) and branch (** ***Olea europaea*** **) with the associated calibrated radiocarbon date.** Shown are olive stones (A–D; F; H), olive wood (G) and cabbage seed (E). The scale for each macro-remain is denoted on the pictures.
(TIF)

Figure S3 **Calibrated calendar age probability distributions for the samples from the Levels 7D and 6E.** The 1σ (68%) and 2σ (95%) confidence levels are respectively indicated by the upper and lower lines under each distribution.
(TIF)

Supporting Information S1 **Gibala-Tell Tweini: details of the Middle-Late Bronze Age (3190±40 ^14^C yr BP) and Iron Age (2845±35 ^14^C yr BP) conflagrations.**
(DOC)
