# Supplementary figures and images for: The Sea Peoples, from Cuneiform Tablets to Carbon Dating
Source: PLoS One. 2011 Jun 8;6(6):e20232. doi: 10.1371/journal.pone.0020232 (PMC3110627; doi:10.1371/journal.pone.0020232)

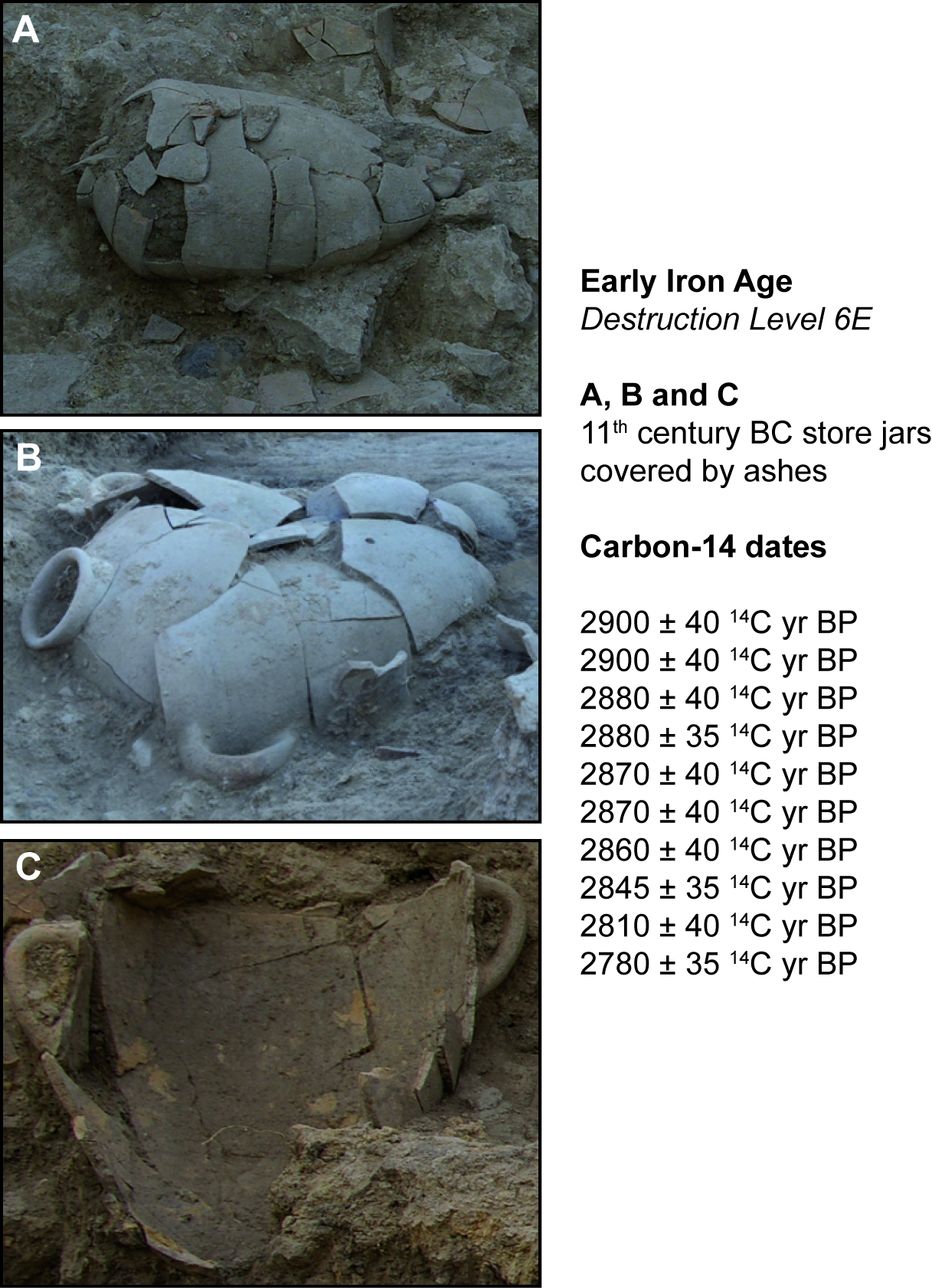

Supplement: Figure S1 — Gibala-Tell Tweini: storage jars found in the Early Iron Age destruction layer. The carbon-14 dating results provide a chronological framework for the Early Iron Age in the Northern Levant. (TIF) [file pone.0020232.s001.tif]

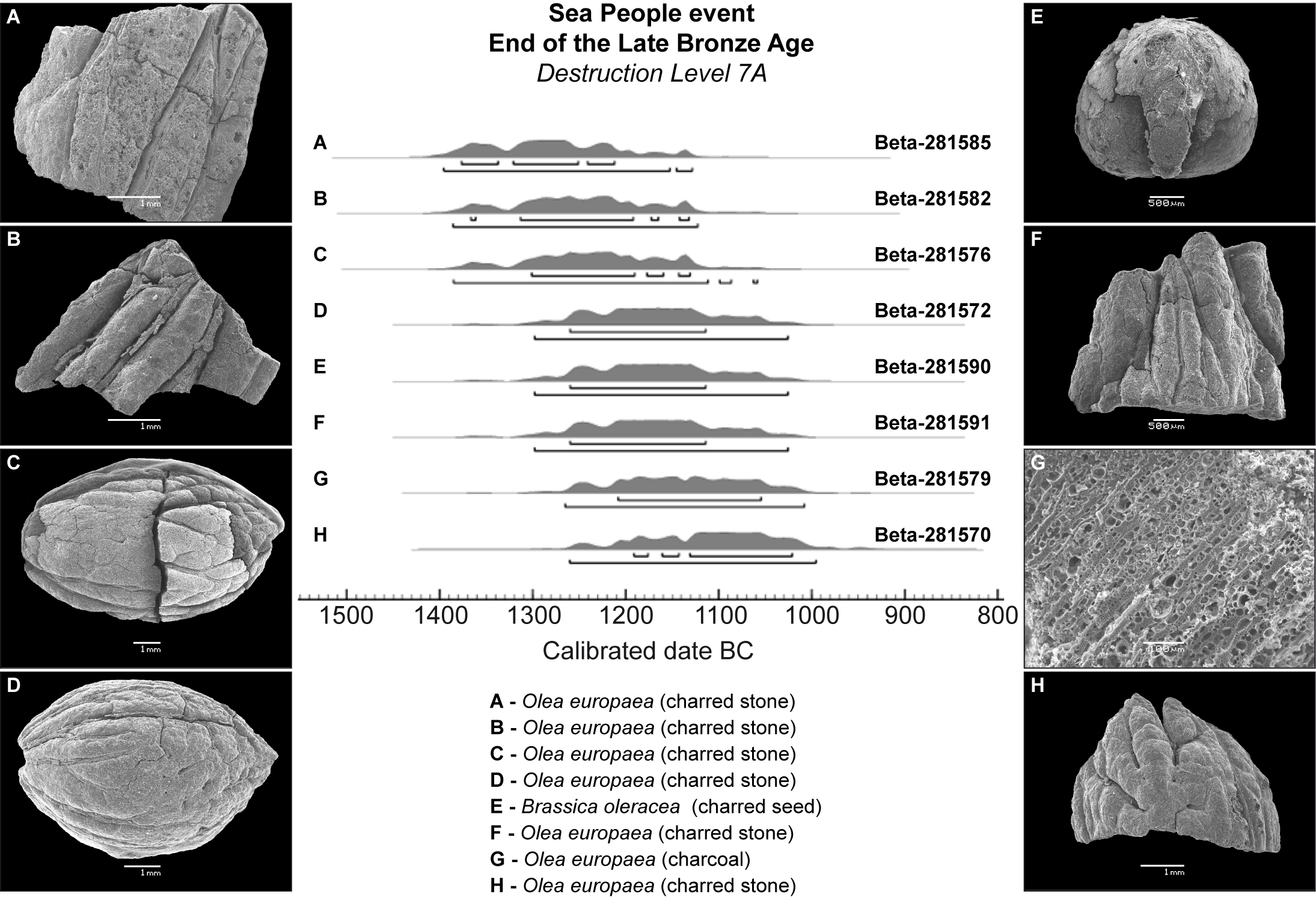

Supplement: Figure S2 — Scanning electron microscopy pictures showing the Sea People event burnt macro-remains of short-lived samples ( Olea europaea , Brassica oleracea ) and branch ( Olea europaea ) with the associated calibrated radiocarbon date. Shown are olive stones (A–D; F; H), olive wood (G) and cabbage seed (E). The scale for each macro-remain is denoted on the pictures. (TIF) [file pone.0020232.s002.tif]

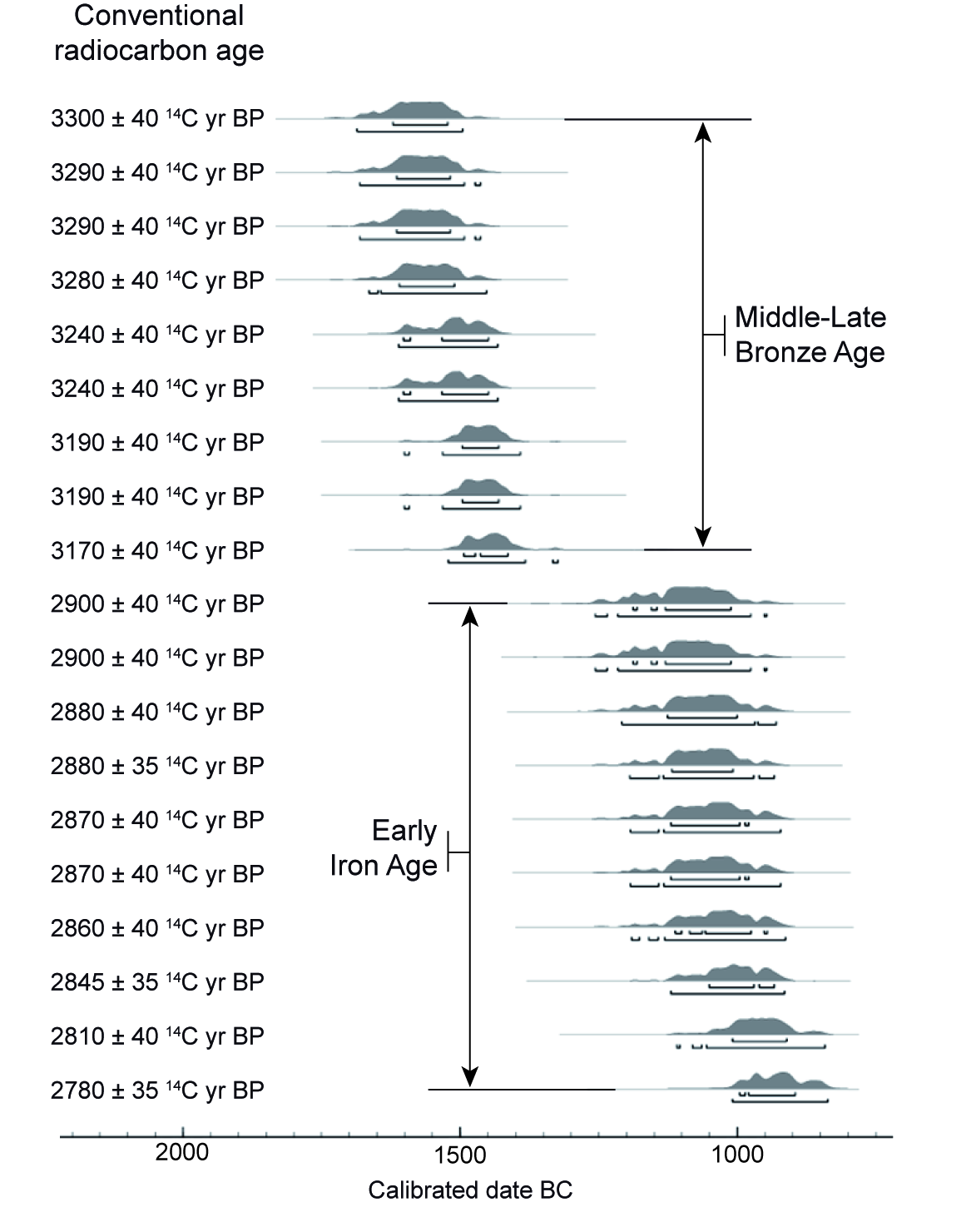

Supplement: Figure S3 — Calibrated calendar age probability distributions for the samples from the Levels 7D and 6E. The 1σ (68%) and 2σ (95%) confidence levels are respectively indicated by the upper and lower lines under each distribution. (TIF) [file pone.0020232.s003.tif]
