# Supplementary material for: The Sea Peoples, from Cuneiform Tablets to Carbon Dating
Source: PLoS One. 2011 Jun 8;6(6):e20232. doi: 10.1371/journal.pone.0020232 (PMC3110627; doi:10.1371/journal.pone.0020232)
Supplement: Supporting Information S1 — Gibala-Tell Tweini: details of the Middle-Late Bronze Age (3190±40 14C yr BP) and Iron Age (2845±35 14C yr BP) conflagrations. (DOC) [file pone.0020232.s004.doc]

**Supporting information S1**

**Early Iron Age** For the Early Iron Age, most of the radiocarbon-based archaeological data come from the southern Levant [35,36,37,38] whilst regions such as Cyprus, Greece and northern Levant lack substantial anchors in absolute chronology. Here, we report the first AMS ^14^C dating of a widespread conflagration layer at Gibala-Tell Tweini. This destruction during the final part of the Early Iron Age I, termed Level 6E, ended a major architectural phase that started soon after the Sea People event. The earliest Iron Age occupation of the 12^th^ century BC (Level 6G-H) is characterized by the reuse of ruins and the construction of new buildings.

The Iron Age conflagration was dated by a set of seven samples retrieved from seven key loci including two-well preserved storage jars found in room contexts (Fig. S1). Three previous samples [10] have been added to the matrix yielding a total of ten samples for this layer (Fig. S3). No weighted average value was calculated. The short-lived sample (*Olea europaea*) serves as a chronological reference, with a conventional radiocarbon age of 2845±35 ^14^C yr BP (1σ: 1050-970 BC, using IntCal09). The 1σ range places the conflagration at the Iron Age I-II transition according to the modified conventional chronology [37].

The ^14^C dating results are consistent with the Cypriot White Painted I ceramics (typologically dated to 1050-950 BC) and the storage jars found *in situ* (typologically dated to the 11/10^th^ century BC) (Fig. S1). No historical and textual evidence from this period is so far available at Gibala-Tell Tweini or other cities in the northern Levant, which could be related to the destruction. A second phase of invasion or regional conflicts is one of the most plausible causes for the conflagration.

**First conflagration** A first conflagration of the harbour town was identified during excavations by an ash layer found in Bronze Age houses. This ash layer was radiocarbon-dated to estimate the time period between the first fire event (Level 7D) and the Sea People event (Level 7A). Nine radiocarbon dates were obtained for this ash layer (Fig. S3). The short-lived sample (*Lens culinaris*) serves as a chronological reference, with a conventional radiocarbon age of 3190±40 ^14^C yr BP (1σ: 1500-1420 BC, using IntCal09). This first fire event is placed at the Middle-Late Bronze Age transition according to the historical chronology (*ca.* 1500 BC).

**Supporting references**

35 Bruins HJ, Van der Plicht J, Mazar A (2003) ^14^C Dates from Tel Rehov: Iron-Age Chronology, Pharaohs, and Hebrew Kings. Science 300: 315-318.

36 Boaretto E, Jull AJT, Gilboa A, Sharon I (2005) Dating the Iron Age I/II transition in Israel: first intercomparison results. Radiocarbon 47: 39-55.

37 Mazar A, Bronk Ramsey C (2008) ^14^C dates and the Iron Age chronology of Israel: a response. Radiocarbon 50: 159-180.

38 Finkelstein I, Piasetzky E (2010) Radiocarbon dating the Iron Age in the Levant: a Bayesian model for six ceramic phases and six transitions. Antiquity 84: 374-385.
